# Situational Analysis of Healthcare and Medical Diagnostic Testing Facility Availability in Selected Blocks of Muzaffarpur District, Bihar, India: A Cross-Sectional Study

**DOI:** 10.7759/cureus.46037

**Published:** 2023-09-26

**Authors:** Pragya Kumar, Shamshad Ahmad, Saurabh Kumar, Naveen K G, Purushottam Kumar, Neeta Kumar, Sanjay Pandey

**Affiliations:** 1 Community and Family Medicine, All India Institute of Medical Sciences, Patna, Patna, IND; 2 Epidemiology and Public Health, All India Institute of Medical Sciences, Patna, Patna, IND; 3 Community and Family Medicine, Maharshi Devraha Baba Medical College, Deoria, Patna, IND; 4 Epidemiology and Public Health, Indian Council of Medical Research, New Delhi, IND

**Keywords:** covid-19 management, situation analysis, accessibility, diagnostic laboratory services, healthcare facility availability

## Abstract

Introduction: This study presents a comprehensive assessment of healthcare facilities, focusing on workforce composition, operational dynamics, diagnostic laboratory services, and accessibility considerations. The comparison between government and private healthcare sectors provides insights into service delivery and potential disparities. The study's rationale, objectives, and methodology are explored in the context of the Indian healthcare landscape.

Methods: A cross-sectional analysis was conducted in Muzaffarpur district, Bihar, targeting selected urban and rural blocks. The study employed geolocation data to analyze accessibility to healthcare facilities. Data collection involved on-site visits, structured questionnaires, and consultation of the Indian Council of Medical Research (ICMR)'s framework. The assessment concentrated on the availability of tests offered by the LaBike platform, and workforce compositions were compared.

Results: Government healthcare facilities exhibited a balanced distribution of doctors, nurses, and grassroot workers, reflecting comprehensive healthcare provisions. Private facilities, although featuring moderate doctor and nurse presence, lacked grassroot workers. Diagnostic test prevalence was evident, with core tests, such as CBC and blood glucose, available in over 85% of facilities. Government facilities provided tests free of charge, while private facilities showcased a diverse cost spectrum. Proposed interventions received strong support from both sectors, indicating the potential for innovative healthcare solutions.

Accessibility analysis: Urban intervention and control sites demonstrated comparable accessibility, with facilities located within 2 km. In rural intervention and control sites, distances varied significantly. Mushahari, a rural intervention site, required participants to travel 6 km to the nearest facility, impacting healthcare access. By contrast, Marwan, a rural control site, featured a shorter distance of 3 km.

Conclusion: This study's comprehensive evaluation of healthcare facilities offers valuable insights into workforce dynamics, diagnostic services, and healthcare interventions in the context of government and private sectors. The findings underscore the significance of addressing workforce gaps and promoting equitable access to diagnostics. By informing evidence-based decision-making, this study contributes to the optimization of healthcare service delivery, aiming to enhance healthcare quality and accessibility for all.

## Introduction

Access to quality healthcare and diagnostics is essential for equitable healthcare delivery [1-3]. This study is a part of the LaBike intervention, aiming to revolutionize healthcare access in underserved areas. LaBike technology [4] stands out as a groundbreaking solution to enhance medical diagnostic testing in resource-constrained areas. Mounted on a bicycle, this mobile diagnostic lab brings testing services to remote populations where conventional labs are unreachable. LaBike's success lies in its ability to improve diagnostic access, reduce result delivery time, and expedite treatment, addressing financial and logistical barriers. It has been proven effective in diagnosing anemia, diabetes, and infectious diseases, promoting timely interventions. LaBike aligns with patient-centered care, catering to local needs, and fosters community engagement.

The LaBike intervention employs technology to bridge healthcare disparities. The objective is to comprehensively assess healthcare facilities and diagnostics in Muzaffarpur district in Bihar, India. By examining and comparing the current manpower and diagnostic tests availability along with their accessibility for the community, this research aims to inform strategies for enhancing healthcare quality. Thus, this study validates the feasibility and disparities in healthcare delivery, focusing on diagnostics. Evaluating both government and private sectors in Muzaffarpur district, the research addresses the need for evidence-based decisions in healthcare provisioning.

The rationale arises from the urgent need to improve healthcare delivery in resource-limited settings. The intricate relationship among the healthcare workforce, diagnostics, and accessibility warrants a careful examination to identify areas for improvement [5]. This study's innovation lies in its holistic approach, encompassing healthcare facilities, diagnostics, and accessibility within a unified framework for deeper insights.

In regions burdened by infectious and non-infectious diseases and constrained healthcare resources, accessing accurate diagnostics poses a challenge due to limited laboratories [6]. Promising innovations, such as point-of-care rapid tests, address this, yet hurdles, including cost and reliability, hinder widespread use. To address these gaps, innovative diagnostic solutions are vital. In rural Peru, medical doctors (MDs) face barriers due to limited point-of-care tools and healthcare system deficiencies. They identify critical diagnostic needs while emphasizing technological innovations' potential, underlining the importance of broader social and policy considerations [7].

Badrick et al. [8] conducted a study in India to evaluate diagnostic laboratory quality, productivity, and reporting efficiency. This investigation highlighted challenges in resource-limited settings and emphasized the need for efficient and accurate test result reporting to improve patient care. The study underscores the importance of innovative solutions, like LaBike technology, to bridge diagnostic gaps for underserved populations. India's implementation of a National Essential Diagnostics List is a significant step toward integrating quality diagnostics into healthcare. This initiative aligns with existing programs, potentially alleviating the financial burden of diagnostics, improving individual and public health outcomes [9].

In rural Bihar, diagnostic facility scarcity compounds broader healthcare challenges, including inadequate infrastructure, sanitation, nutrition, and essential medicines access, intensifying health risks. Innovative global approaches have emerged to tackle diagnostic availability challenges, especially in regions like India. Leveraging technology and creative solutions, strategies, such as telemedicine and mobile health (mHealth) interventions, have expanded diagnostic reach to remote areas. These tools enable remote data collection and transmission for central analysis, enhancing access and early disease management [10]. Point-of-care testing (POCT) revolutionizes diagnostics by providing rapid results, particularly effective in resource-limited settings. Studies show POCT's efficacy in diagnosing infectious diseases and diabetes, leading to faster treatment initiation [11]. Public-private partnerships (PPPs) bolster diagnostic access through collaborations between governments, private sector entities, and non-governmental organizations (NGOs), enhancing infrastructure and coverage, ultimately improving health outcomes [12].

In conclusion, inadequate diagnostic facilities in regions like Bihar adversely impact health outcomes and exacerbate burdens on marginalized communities. Addressing this requires urgent attention to bolster diagnostic infrastructure and ensure equitable access to quality healthcare services.

Inadequate diagnostics impact health outcomes, necessitating improved infrastructure for equitable healthcare access. This study is a pivotal step in the LaBike intervention, addressing healthcare disparities for underserved populations.

## Materials and methods

The primary objective of this cross-sectional study was to conduct an extensive situational analysis of healthcare and medical diagnostic testing facilities within four specific blocks of Muzaffarpur district, Bihar. This study was a critical component of a broader research initiative entitled "Task Force Study for Evaluation of Community Level Acceptability, Scalability, and Linkage within the Health System of ICMR Pre-validated Labike Technologies for Screening and Diagnosis in Rural & Urban Populations: An Implementation Research." A brief project summary can be found in the Appendix section.

The selection of the four specific blocks in Muzaffarpur district was based on several key considerations: The first one is the large disadvantaged populations.* *Two of the selected blocks, namely, Mushahari and Balughat, were designated as intervention sites. These areas were chosen because they were characterized by a significant concentration of disadvantaged populations. This demographic profile made them particularly relevant for the study's objectives, as understanding healthcare and diagnostic testing facilities in areas with higher levels of vulnerability is crucial for addressing health disparities and improving healthcare delivery.

The second consideration is logistical convenience.* *Another significant factor in the selection of the four blocks was the presence of ongoing projects in the same Muzaffarpur district. The fact that your research team already had existing projects in the area provided a practical advantage. It streamlined logistics, facilitated access to local resources, and allowed for the efficient use of existing infrastructure and relationships with local stakeholders. This synergy between projects can enhance the overall effectiveness and efficiency of research initiatives.

The third one is control blocks for research validity. Marwan and Kannauli were chosen as control blocks, which were situated at a 5-10 km distance from the intervention sites. This geographical separation was essential to prevent contamination, ensuring that the intervention's effects did not spill over into the control areas.

Muzaffarpur, Bihar, India, housed 393,724 individuals in the 2011 census, with 52.96% males and 47.04% females. Literacy rates were 74.74%, with male literacy at 77.99% and female literacy at 71.08%. Religious diversity emerged with 275,233 Hindus, 74,680 Muslims, and 1,352 Christians. The population split to 9.9% in urban and 90.1% in rural areas. Urban literacy was 80.2%, rural literacy was 61.5%, and sex ratio was at 889 (urban) and 901 (rural). Muzaffarpur's overall literacy stood at 63.43%. Male literacy was 58.84%, while female literacy was 44.96%. Census data noted 751,975 scheduled caste and 5,979 scheduled tribe individuals, revealing social dynamics [13].

The primary objective of this specific study was to assess the healthcare and medical diagnostic testing facilities in the selected blocks by examining their healthcare workers and diagnostic test availability along with their accessibility for the community. The study duration spanned from September 5 to September 12, 2022. Marwan and Mushahari blocks represented rural sites, while Balughat and Kanhauli were urban sites.

The data collection process began at the government health facility within each block, extending outward to encompass all healthcare and laboratory testing facilities. Our field team comprised a medical officer (Scientist C Grade), a social worker cum data entry operator, and four lab technicians. On-site visits were conducted to health facilities and diagnostic centers. The data collection procedure was facilitated through the structured data entry sheets provided in Appendix A and Appendix B. This sheet encompassed the checklist and items enquiring various details about the health facilities and of the diagnostic services offered. Data collection was efficiently carried out using this sheet through the Epicollect5 platform.

Ethical approvals were diligently obtained from the Institutional Research Committee (approval no. AIIMS/IRC/2020/736) and Institutional Ethical Committee (approval no. AIIMS/IEC/2020/736) of All India Institute of Medical Sciences (AIIMS), Patna. The overarching goal of this situational analysis aligns with the broader study's objective, which aims to assess the feasibility of implementing LaBike technologies in both rural and urban contexts, with a focus on enhancing accessibility and healthcare services for underserved populations.

## Results

Healthcare facilities

**Figure 1:**
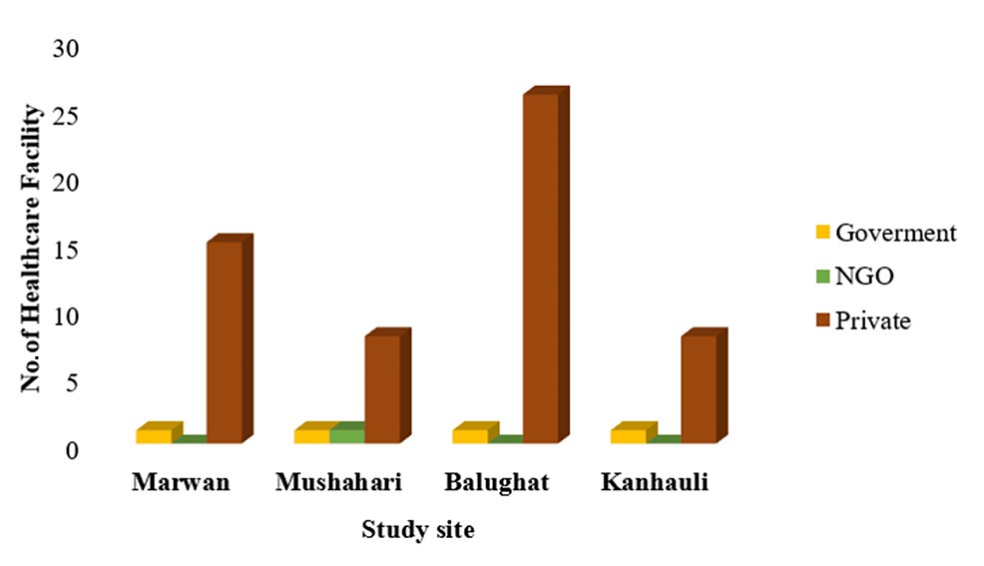
Health facility availability and their type (N = 62) NGO: non-governmental organization The graph was created in Microsoft Word by the authors.

**Figure 2:**
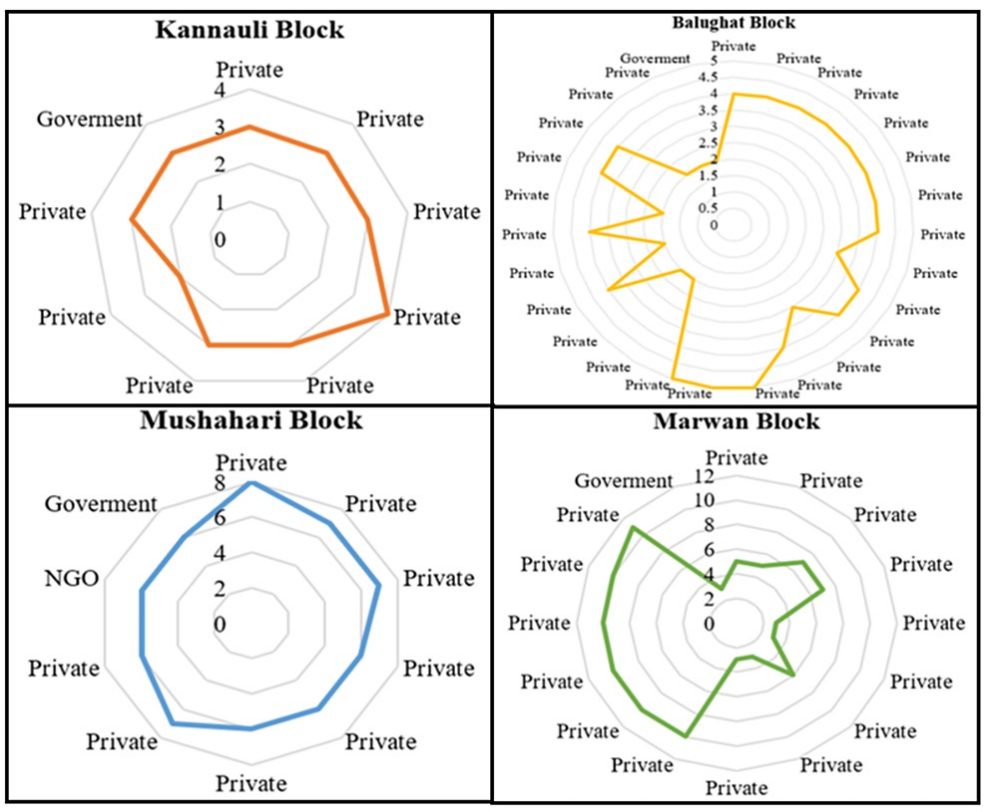
Accessibility of healthcare facilities NGO: non-governmental organization The graph was created in Microsoft Word by the authors.

Geolocation, the precise determination of geographic coordinates using GPS technology, played a vital role in our study. We utilized geolocation utilizing Google Maps to investigate the accessibility of healthcare facilities within specific block territories. By capturing the health facility's location during the team visit and identifying prominent landmarks in the baseline data collection site (of the main study) of the block with latitude and longitude coordinates, we could calculate the distances between these landmarks and healthcare facilities. Basically, here, we considered this landmark as a proxy for the residential locations of the study participants (of the main study's baseline survey) and the distance in kilometer as a proxy for the accessibility from the site. This data provided crucial insights into healthcare accessibility, helping identify areas that may require improved infrastructure or transportation options to enhance access for residents. Figure 1 depicts that the rural blocks Marwan and Mushahari each had one government health facility, alongside varying numbers of private health facilities (15 and 8, respectively). A radar diagram (Figure 2) was employed to visualize these distance differences.

It is noteworthy that Mushahari also accommodated a solitary NGO-operated health facility. Within Mushahari, the minimum distance to reach a healthcare facility from the data collection area was 6 km, which led to the community health center (CHC) of the block, along with five out of the eight private facilities and the sole NGO-run healthcare facility. In Marwan, the minimum distance was 3 km, with a primary healthcare centre (PHC) of the block and four other private healthcare facilities.

Balughat had a government health facility and a substantial number of private facilities (26). The minimum distance to the nearest healthcare centre was identical at 2 km for both private (six facilities) and the PHC of the block. Similarly, Kannhauli accommodated one government health facility and eight private facilities, with the nearest health center being a private facility within 2 km. The PHC of the block was situated at a distance of 3 km from the residences of the participants.

The composition of health facility workforces varied notably (see Table 1). Health facility types were associated with distinct workforce compositions. Government facilities exhibited an average of 4 ± 3 doctors, with a median of 21 nurses (interquartile range: 6-38) and a median of 59 grassroot workers (interquartile range: 8-144). NGO facilities had one doctor, no nurses, and two grassroot workers. Private facilities featured an average of 2 ± 1 doctors, a median of one nurse (interquartile range: 1-3), and lacked grassroot workers.

**Table 1:** Existing work force of the health facilities NGO: non-governmental organization *As there was only one NGO-run health facility, the number of working days have been quoted directly. **All figures are represented as mean ± standard deviation (SD) as they are normally distributed. ***All figures are represented as median (interquartile range) as the data are skewed.

Health facility type	Work force		
	Doctors**	Nurses***	Grassroot workers***
Government	4 ± 3	21 (6 – 38)	59 (8 – 144)
NGO*	1	0	2
Private	2 ± 1	1 (1 – 3)	0

**Figure 3:**
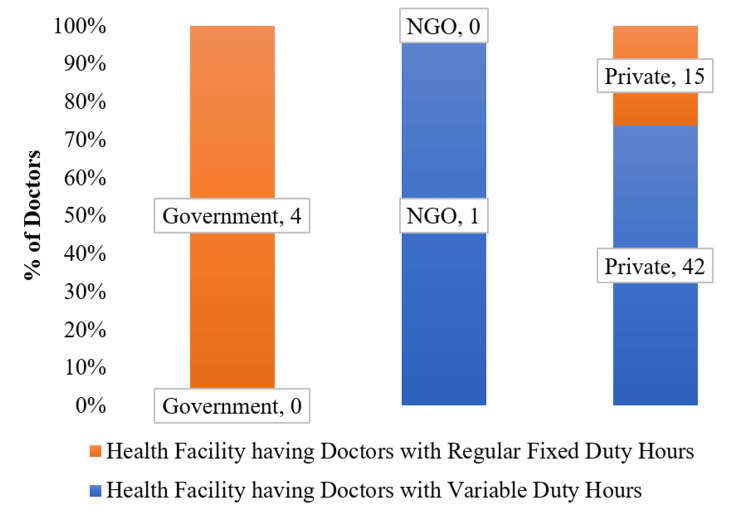
Work pattern of the doctors in the health facilities NGO: non-governmental organization The graph was created in Microsoft Word by the authors.

As shown in Figure 3, in government facilities, the doctors were primarily posted on regular fixed duty hours, while private facilities predominantly (nearly 73%) adopted variable duty hours. Notably, the single NGO-operated healthcare facility in Mushahari block had only one doctor with variable duty hours.

Table 2 illustrates the mean working days per week for government, NGO-run, and private healthcare facilities across all four blocks. The values indicate that both the government and private facilities had an average of six working days per week with some amount of variability (SD 0.5 and 1.5). The NGO-operated facility in Mushahari had five working days per week.

**Table 2:** Working days per week NGO: non-governmental organization All figures are represented as mean ± standard deviation (SD). *As there was only one NGO-run health facility, the number of working days has been quoted directly.

Government	NGO	Private
6 (0.5)	5*	6 (1.5)

Table 3 indicates that the government health facilities had a median total bed capacity of eight (with an interquartile range of 2-18) and allocate a median of three beds (with an interquartile range of 2-4) for COVID-19 patients. In comparison, NGO-operated facility had a total bed capacity of only four, while none of the private facilities had in-patient facilities.

**Table 3:** Bed capacity (total and COVID-19) of health facilities NGO: non-governmental organization All figures are represented in median (interquartile range). *As there was only one NGO-run health facility, the total bed capacity has been quoted directly.

Health facility type	Total bed capacity (median (IQR))	Beds dedicated for COVID-19 patients (median (IQR))
Government	8 (2–18)	3 (2–4)
NGO	4*	0
Private	0	0

While all the government and very few private (10) health facilities provided additional training for staff in COVID-19 management, the majority of private (47) along with NGO-run health facilities had no such training (Table 4).

**Table 4:** Additional training for staff in COVID-19 management NGO: non-governmental organization All figures have been quoted along with respective percentages (n'(%)).

Health facility type	Additional training for staff in COVID-19 management	
	Yes	No
Government (n = 4)	4 (100%)	0
NGO (n = 1)	0	1 (100%)
Private (n = 57)	10 (17.5%)	47 (82.4%)

During the survey of the health facilities, the team checked for the availability of various diagnostic tests, including COVID-19, hemoglobin, blood sugar, lipid profile, kidney profile, liver function test (LFT), serum electrolytes, hormones, X-ray, ultrasound sonography (USG), CT scans, and PET scans. This list of tests was thought to serve as a proxy for routine and essential tests and special tests (e.g., hormones and PET scan). Here, no Indian Public Health Standards (IPHS), National Accreditation Board for Hospitals (NABH), or any other national and international guidelines or checklist was utilized for the assessment due to many operational constrains. This list was confirmed and finalized by department experts along with project stakeholders from the Indian Council of Medical Research (ICMR).

Table 5 explains the following details: NGO-run health facility lack in all diagnostic tests. In the realm of COVID-19, three (75%) government facilities and one (1.7%) private facility conducted antigen testing. Routine tests, such as hemoglobin and blood sugar, were carried out in all (100%) government facilities, whereas only in 24 (42%) and 36 (63%) of private facilities. Kidney profile was available in three out of four government health facilities, while liver profile, electrolyte, and hormone tests were available only in one (25%) government health facility. The availability of these tests in private facilities was scattered in distribution. X-ray scans were present only in one (25%) government and 2 (3.5%) private health centers. Meanwhile, government health centers lacked USG, and three (5.2%) of the private clinics had USG facilities. Notably, none of the health facilities had CT and PET scans.

**Table 5:** Availability of various diagnostic tests in health facilities NGO: non-governmental organization; USG: ultrasound sonography; CT scan: computed tomography scan; PET scan: positron emission tomography scan All figures have been quoted along with respective percentages (n'(%)).

Diagnostic tests	Government (n = 4)	NGO (n = 1)	Private (n = 57)
Covid-19 testing	3 (75%)	0	1 (1.7%)
Hemoglobin	4 (100%)		24 (42%)
Blood sugar	4 (100%)		36 (63%)
Lipid profile	3 (75%)		7 (12%)
Kidney profile	3 (75%)		8 (14%)
LFT	1 (25%)		8 (14%)
Serum electrolytes	1 (25%)		5 (9%)
Hormones	1 (25%)		1 (1.7%)
X-ray	1 (25%)		2 (3.5%)
USG	0		3 (5.2%)
CT scan	0		0
PET scan	0		0

In reference to Table 6, except for the emergency and essential medicines, NGO-run health center lacked all other facilities, while the medicines were available in all government and a significant number of private facilities (44 out of 57). Two out of four government facilities were equipped with an isolation ward with and without oxygen support, ambulance service with advanced life support (ALS), and separate functional operation theater (OT) for COVID and non-COVID emergencies.

**Table 6:** Availability of infrastructure for COVID-19 management NGO: non-governmental organization; ALS: advanced life support; ICU: intensive care unit; OT: operation theater All figures have been quoted along with respective percentages (n'(%)).

Diagnostic tests	Government (n = 4)	NGO (n = 1)	Private (n = 57)
Emergency and essential drugs	4 (100%)	1 (100%)	44 (77%)
Isolation ward without oxygen support	2 (50%)	0	6 (10.5%)
Isolation ward with oxygen support	2 (50%)	0	4 (7%)
Intubation kit	1 (25%)	0	0
Functional ventilator/ICU for COVID patients	0	0	0
Functional ventilator/ICU for non-COVID patients	0	0	0
Ambulance facility with ALS	2 (50%)	0	3 (5.2%)
Separate functional OT for COVID emergencies	2 (50%)	0	0
Functional OT for non- COVID emergencies	2 (50%)	0	9 (16%)

Table 7 displays the acceptance of the proposed interventions from the main study within various health facility categories. Mobile Lab was the concept of LaBike, and Home Health Guide (HHG) was a new cadre to be created in the main study, which was completely based on the concept of community involvement. HHG will be a link between the beneficiaries and health system. Notably, all government facilities (n = 4) embraced the Mobile Lab, Health Diary, and HHG interventions. Health ID Health Data Portal was a concept of universal health ID and Electronic Medical Record (EMR) portal to be created. This intervention is planned to be modified. Instead of a new health ID and EMR portal concept, Ayushman Bharat Health Account (ABHA) number is planned to be utilized in the main study. However, in this situational analysis, we adhered to the old proposal and assessed the acceptance of the four already proposed interventions. In the private sector, a substantial proportion of facilities (57 out of 57) accepted the Mobile Lab, while 41 out of 57 (72%) adopted the Health Diary and 24 out of 57 (42%) embraced the HHG. Conversely, the NGO-operated sector displayed no acceptance for any of the interventions. Acceptance of the Health ID Health Data Portal under the study was seen in two (50%) government facilities, with no uptake observed in the NGO-operated or private facilities. These findings underscore differential acceptance levels for the proposed interventions among government and private health facilities.

**Table 7:** Acceptance of interventions proposed in the main study for the health facilities NGO: non-governmental organization All figures have been quoted along with respective percentages (n'(%)).

Proposed interventions	Government (n = 4)	NGO (n = 1)	Private (n = 57)
Mobile Lab	4 (100%)	0	57 (100%)
Health Diary	4 (100%)	0	41 (72%)
Home Health Guide (HHG)	4 (100%)	0	24 (42%)
Health ID Health Data Portal under the study	2 (50%)	0	0

Medical laboratory facilities

**Figure 4:**
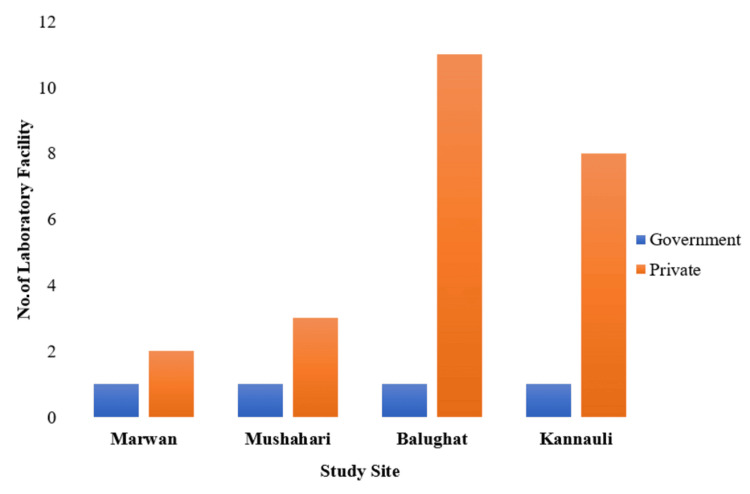
Laboratory facility available and their type (N = 28) The graph was created in Microsoft Word by the authors.

The distribution of laboratory types across the study sites unveiled distinct trends (Figure 4). Each site had a single government-operated laboratory as a component of the public health facility. Private laboratories showed variability: Marwan and Mushahari each had two and three, respectively, while Balughat and Kannauli boasted higher numbers with 11 and eight, respectively.

**Figure 5:**
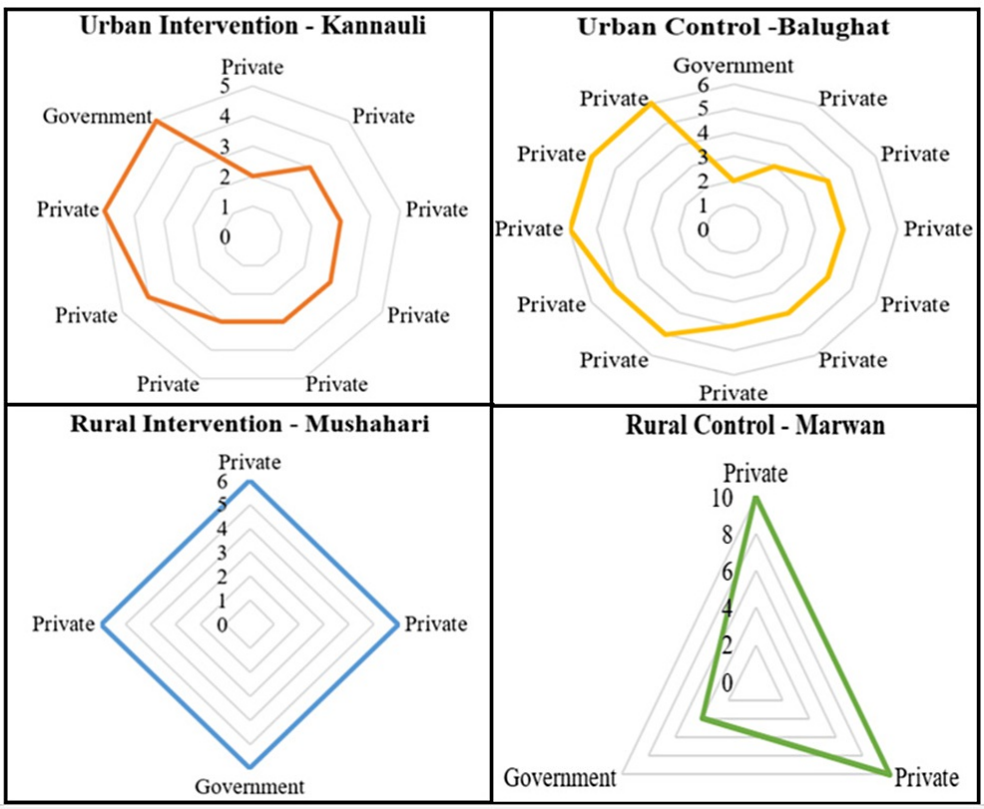
Accessibility of medical diagnostic testing facilities The graph was created in Microsoft Word by the authors.

The evaluation of accessibility to laboratory facilities was conducted using geolocation data, providing valuable insights into the spatial distribution of healthcare resources. In the urban intervention site of Kannauli, the nearest laboratory facility, a private establishment, was situated at a distance of 2 km from the common landmark (Figure 5). Similarly, in the urban control site of Balughat, the nearest testing facility, a government-operated urban primary health center (UPHC), was also located at a distance of 2 km. In the rural intervention site of Mushahari, participants had to cover a distance of 6 km to reach the nearest laboratory, which was available in both government and private sectors. Lastly, in the rural control site of Marwan, the closest testing facility, a government-operated UPHC, was situated at a distance of 2 km.

During the survey, the team checked the availability of tests that was proposed to be incorporated in LaBike by ICMR in their protocol of the main study, which include CBC, blood glucose, lipid profile, kidney function test (KFT), LFT, erythrocyte sedimentation rate (ESR), urine routine, HIV, hepatitis B surface antigen (HBsAg), Widal, rapid malaria, glycosylated hemoglobin (HbA1C), and thyroid profile. Detailed data can be found in Appendix C.

All the tests are available in the government health centers being free of cost for the public. Table 8 primarily represents the mean cost of all the tests included in survey in the private laboratories.

**Table 8:** Availability and mean cost of tests checked for availability (N = 32) *Average cost has been quoted.

Tests	Availability (out of 32 labs)	Mean cost* (in Indian Rupees)
Complete blood count (CBC)	28 (87.5%)	264
Blood glucose	28 (87.5%)	68
Lipid profile	27 (84.4%)	503
Kidney function test (KFT)	27 (84.4%)	605
Liver function test (LFT)	27 (84.4%)	588
Erythrocyte sedimentation rate (ESR)	26 (81%)	100
Urine routine	25 (78%)	207
Human immunodeficiency virus (HIV)	16 (50%)	203
hepatitis B surface antigen (HBsAg)	14 (43.7%)	182
Widal	16 (50%)	188
Rapid malaria	17 (53%)	300
Glycosylated hemoglobin (HbA1C)	18 (56%)	600
Thyroid profile	15 (47%)	462

## Discussion

The comprehensive evaluation of healthcare facilities has yielded significant insights into workforce composition, operational dynamics, diagnostic laboratory services, and accessibility considerations, with a specific emphasis on comparing government and private healthcare sectors. The rationale underlying the selection of specific assessment parameters, along with the distinctive methodology employed for diagnostic laboratories and accessibility analysis, merits further exploration.

The choice of parameters for assessment was guided by the study's objectives and the questionnaire provided by the ICMR for situational analysis. While the questionnaire served as a foundational framework, it underwent tailored modifications to suit the unique characteristics of the study sites. Evaluation of both healthcare and medical diagnostic testing facilities focused on the availability of tests present in the LaBike platform. This approach aligned with the study's goal of assessing the feasibility and readiness for implementing diagnostic services offered by LaBike.

The decision to consider only tests present in LaBike was a strategic one, enabling a focused assessment of the healthcare landscape's preparedness to integrate these specific diagnostic services. This approach aimed to provide insights into the adaptability and potential implementation of the LaBike platform within the existing healthcare infrastructure. Importantly, the study encompassed a comprehensive list of all tests available in each laboratory, including their associated costs. This dual approach examining both the presence of LaBike-listed tests and the broader range of available tests enhanced the study's depth and scope.

Notably, the comparison between government and private healthcare facilities reveals significant disparities in workforce compositions, indicative of distinct approaches to healthcare service delivery. Government facilities display a balanced distribution of doctors, nurses, and grassroot workers, reflecting a comprehensive approach to healthcare provision that manages medical, nursing, and community-based aspects efficiently. By contrast, private facilities exhibit a different workforce pattern, with a moderate presence of doctors and nurses but a notable absence of grassroot workers. This discrepancy underscores the importance of addressing these gaps, particularly in private healthcare settings, to ensure holistic healthcare services.

The study also highlights variations in doctors' work patterns across facility types. Government facilities tend to operate with variable duty hours, reflecting flexibility in patient care management, while private facilities predominantly adhere to fixed duty hours. This variation may mirror differences in patient load, operational structures, and service extent.

Moreover, the assessment of total bed capacities and beds designated for COVID-19 patients offers insights into healthcare facility readiness during healthcare crises. Government facilities possess a higher median bed capacity, indicating the potential to accommodate larger patient influxes. By contrast, private facilities lack in-patient beds entirely, suggesting potential limitations in handling patients requiring hospitalization. This finding underscores the necessity for exploring the capacity and scope of private healthcare establishments, particularly in emergencies.

Accessibility analysis

The evaluation of accessibility to healthcare facilities was a pivotal aspect of this study. In the urban intervention site of Kannauli, the participants had access to a private healthcare facility within a mere 2 km distance, while the government-operated UPHC in Balughat, the urban control site, was equally accessible at the same distance. In the rural intervention site of Mushahari, accessibility to healthcare was a challenge, as the participants needed to cover a substantial distance of 6 km to reach the nearest laboratory, available in both government and private sectors. Similarly, in the rural control site of Marwan, the closest testing facility, a government-operated UPHC, was situated at a distance of 2 km. These accessibility variations emphasize the need for targeted efforts to bridge healthcare access gaps in underserved regions.

Turning to the cost of diagnostic tests, a compelling contrast emerges between government and private healthcare facilities. Government facilities offer all tests free of charge, aligning with principles of accessible and affordable healthcare. Conversely, private facilities present a diverse cost spectrum for tests, making determination of an average cost challenging. 

The consensus of healthcare facilities with the proposed interventions outlined in the main study signals a positive response to innovative healthcare solutions. Government facilities unanimously support the proposed interventions, including the Mobile Lab, Health Diary, HHG, and Health ID Health Data Portal. Similarly, private facilities exhibit strong consensus, with a substantial majority of 57 out of 57 facilities endorsing the Mobile Lab intervention and 41 out of 57 expressing acceptance for the Health Diary. In addition, 24 private facilities expressed interest in implementing the HHG. While the Health ID Health Data Portal garnered limited agreements, varying perceptions and priorities within different healthcare settings may influence this response. Overall, this alignment underscores the potential for the successful implementation of these interventions, potentially leading to enhanced healthcare accessibility and patient engagement.

## Conclusions

The comparative assessment of diagnostic laboratory services across government and private healthcare facilities has unveiled critical aspects of healthcare provisioning. The approach to selecting assessment parameters, particularly focusing on tests present in LaBike, underscores the study's targeted objectives and methodology. The findings enrich the understanding of healthcare service distribution, priorities, and potential disparities, with implications for informed healthcare planning, policy formulation, and strategies aimed at equitable and accessible diagnostic services for diverse populations.

Throughout the assessment, the research team encountered a challenge in obtaining information from diagnostic laboratories, possibly due to concerns about LaBike as a competitor. This underscores the importance of effective communication and transparency to address potential misconceptions and establish collaborative relationships within the healthcare ecosystem.

While valuable, the study has limitations. Its scope was limited to specific sites, and the cross-sectional data collection approach may restrict generalizability and trend identification. Focusing on workforce availability, rather than comprehensive infrastructure evaluation, limits holistic assessment. In addition, while resource availability was examined, the study did not directly assess accessibility, including out-of-pocket expenditure. Similarly, while exploring test prevalence, broader perspectives encompassing test availability and affordability.

## Appendices

Protocol of the main study: the ICMR Task Force study project

Title of the Project: Task force study for evaluation of community level acceptability, scalability and linkage within health system of ICMR pre-validated LaBike/Technologies for screening & diagnosis in rural & urban population-An implementation research

The project aims to assess the feasibility and effectiveness of implementing the Indian Council of Medical Research (ICMR) pre-validated technologies for healthcare screening and diagnosis through a mobile lab system (LaBike) in both rural and urban communities. The project is led by Dr. Jugal Kishore, Director Professor, and head, of the Department of Community Medicine, Vardhman Mahavir Medical College, Safdarjung Hospital, Delhi.

The project involves collaboration with experts, government bodies, and medical institutions. The coordinating center is the ICMR Headquarters, with an advisory group and data safety monitoring board constituted at ICMR to provide guidance and oversight. The study focuses on bridging gaps in health information management, testing the acceptability of doorstep healthcare services using LaBike, and assessing the cost-effectiveness of these services. The study also aims to evaluate the need for capacity building in preventive health education and health data updating.

The primary objectives include situational analysis of medical facilities, evaluating acceptability and cost effectiveness of LaBike services, validating referral linkages, and assessing the needs for capacity building. The study intends to address the gaps identified in health information management systems and strengthen tools and linkages at the grassroot level. The project aligns with the Ayushman Bharat Digital Health Mission (ABDM) and Ayushman Bharat-Pradhan Mantri Jan Arogya Yojana (PMJAY) to improve healthcare accessibility and data sharing. The study emphasizes the importance of individual health IDs, health records, and digital health systems to enhance healthcare delivery and data availability.

The project will be conducted at various sites across India, including Kashmir, Guwahati, Bhopal, Chennai, Gujarat, Bihar, and Uttar Pradesh. The objective is to evaluate the effectiveness of interventions, such as LaBike services, teleconsultations, and health information management, in improving healthcare delivery and data sharing in remote and underserved areas.

Review of literature

In the existing knowledge review, international practices, such as the Centers for Disease Control and Prevention (CDC)'s health ID program, were mentioned, but it was highlighted that India faces unique challenges due to factors, such as literacy, technology access, and manpower availability. Preliminary works had been carried out, including validation of the LaBike technology and testing the acceptability of health IDs. The need for skilled manpower, lab facilities, and effective health data management were identified as gaps in the existing system.

Several studies from India highlighted deficiencies in healthcare infrastructure, workforce, and equipment at the grassroot level. The Ayushman Bharat scheme was discussed, which aims to provide essential healthcare services but may still leave gaps in remote and underserved areas. The importance of community participation and locally adapted methods for implementation was stressed. The study's design was explained, involving a cluster randomized controlled approach with pre- and post-intervention surveys, cohort follow-up, and qualitative assessments. The study sites were selected to represent different regions across India, and the sample size calculation was discussed, considering factors, such as chronic diseases, prevalence rates, and desired effect sizes. A total sample size of 35,000 participants across seven sites was proposed, with subgroup analyses for intervention effects and rural-urban differences, among others. The study participants would include all family members of participating families, with consent obtained from the head of each family. The selection of districts, towns, or villages for the study would be based on the probability proportional to size (PPS) method, considering both population size and logistical feasibility.

Methodology

The project's methodology is thoughtfully structured, commencing with site selection using the PPS method, ensuring balanced representation. Ethical clearances and funding formalities are diligently addressed. Staff recruitment and orientation, along with LaBike technology placement and Home Health Guide (HHG) training, establish the project's workforce.

Data collection and implementation encompass the distribution of health diaries to individuals with health IDs, baseline blood sample collection using LaBike, and monthly updates facilitated by HHGs and lab technicians. Health data collected monthly are entered into an online platform, facilitating data analysis and timely sharing with local health authorities for informed decision-making.

The end-of-term assessment involves surveys to gauge health status changes, expenses, and community experiences. Qualitative assessments capture intervention feedback and user experiences. Outcome analysis evaluates HHG performance, healthcare expenses, and government scheme utilization between study and control groups.

The project fosters collaboration with local stakeholders and integrates with existing government health programs to enhance healthcare accessibility. Notably, the project integrates with the Ayushman Bharat Digital Health Mission, employs a mixed-method approach for comprehensive analysis, and aspires to break the cycle of poor health literacy and inequalities through community engagement and data-driven interventions.

Throughout a 12-month follow-up period, the project team conducts assessments, meetings, data collection, and analysis, leveraging LaBike technology and health records to drive its objectives, with the potential to significantly elevate healthcare delivery and health outcomes within the communities served.

Mobilization and people management

The Mobilization and People Management section of the proposed health project outlines key strategies and processes for effectively managing personnel and community engagement. These elements are crucial for the successful implementation of the project:

Training for Volunteers and Health Guides

Local youth and school teachers will receive training to become volunteers who will take on roles as health guides. The training curriculum will focus on preventive health measures, the importance of health check-ups, and the responsibilities of health guides. The Project Manager will compile workshop materials, including intervention guidelines and information, education, and communication (IEC) materials. In addition, lab technicians will undergo training covering biochemistry, blood collection techniques, testing methods, and blood tests.

Integration with Government Programs

The project will strategically align itself with government programs that address non-communicable diseases, such as anemia, malaria, and tuberculosis. This alignment aims to leverage existing resources and infrastructure to achieve enhanced health outcomes.

Lab Tests on LaBike

A comprehensive list of tests, including hemoglobin, glucose, and cholesterol, will be conducted using the LaBike technology. The associated costs for these tests will be detailed to ensure transparency in healthcare services.

Target Population Segmentation

The study population will be systematically segmented based on various demographic factors, including age, gender, and education level. This segmentation will enable tailored healthcare interventions.

Capacity Building and Training

Health guides will undergo comprehensive training through both correspondence and practical workshops. This training encompasses an Indira Gandhi National Open University (IGNOU) course, a certified yoga program, and emergency cardiac care training, enhancing their proficiency in delivering healthcare services.

Data Collection and Analysis

Health diaries will be distributed to collect pertinent health-related data from the community. Data analysis will focus on evaluating the utilization and acceptance of interventions, assessing disease burdens, treatment patterns, needs assessments, and more. This approach ensures a data-driven approach to healthcare.

Community Advisory Board and IEC

Community advisory boards will play a pivotal role in fostering communication and motivating individuals to use health diaries. An extensive IEC strategy, incorporating mass media, leader training, and one-on-one counseling, will be employed to engage and educate the community effectively.

Data Safety and Auditing

Robust data safety measures will be implemented, including the secure storage of forms and documents, utilizing only patient IDs for data management. Data auditing procedures will be enforced to ensure data completeness and consistency.

Report Writing and Dissemination

Yearly draft reports will be meticulously prepared and disseminated to stakeholders for feedback. Dissemination meetings, featuring policymakers, program managers, experts, and researchers, will facilitate comprehensive discussions and informed decision-making.

This comprehensive approach to mobilization and people management underscores the project's commitment to community engagement, data integrity, and capacity building, all of which are essential pillars in the endeavor to enhance healthcare accessibility and outcomes within the targeted communities.

Summary of the study material

The study focuses on implementing a health data collection and management system using a mobile lab called LaBike. The mobile lab will provide health services to various age groups and communities, aiming to improve preventive healthcare and health data collection. The project involves training local youth and school teachers to act as volunteers to provide health guidance and preventive health services. LaBike will be equipped to conduct various health tests, and the project aims to collaborate with government programs for non-communicable diseases.

Translation of the Study Material

All study materials, including the protocol, manuals, data collection instruments, and consent forms, will be translated into the local language at each study site to ensure comprehension and accuracy. Back translation to English will be conducted to verify the accuracy of translations.

Previous Validation Studies

A validation study was conducted using LaBike to assess its sensitivity and specificity against gold standard methods. The study involved various health parameters and tests, including biochemical, hematological, and serological tests. The results indicated that LaBike provided accurate results for several parameters.

Publications of Investigators

The investigators have published several papers related to health data collection, community participation, and health planning. These publications demonstrate their expertise in the field and provide context for the current study.

Budget and Resource Allocation

The study's budget includes funding for personnel, training, materials, meetings, communication, and overhead costs. The budget is distributed per site and per year, considering the expenses required for successful implementation.

Data entry sheets

Appendix A. Health Facility Details

**Table 9:** Health facility details

Questions	Options/answers
Study site	Urban – Intervention (Kanhauli) Urban – Control (Balughat) Rural - Intervention (Mushahari) Rural - Control (Marwan) Health Facility Name
Health facility type	Private, government, NGO, others
Health facility address	
Health facility's contact number	
Doctor/MOIC name	
Doctor's contact number	
Duty days/week	1, 2, 3, 4, 5, 6, 7
Duty hours	Fixed variable
How many doctors are available in this health facility?	
How many nursing staff are available in this health facility?	
How many grassroot workers/field staff are available in this health facility?	
How many technicians (Lab, X-ray, USG, other technicians) are available in this health facility?	
What is the total bed capacity of the facility?	
What is the bed capacity of the facility to manage patients affected by COVID-19?	
Does this facility have a functioning cold chain capacity and other arrangements in place to support vaccinations?	Yes, No
Is additional training and support being provided to healthcare workers (in the context of COVID)?	Yes, No
Does this facility have the necessary diagnostic equipment and supplies for:	COVID-19 testing, hemoglobin, blood sugar, lipid profile, kidney profile, liver function test, serum electrolytes, hormones, X-ray, USG, CT scan, PET scan None
Does this facility have the necessary medicines and medical supplies for the management of COVID-19 and other types of patients?	Functional ventilator/ICU for COVID patients/functional ventilator/ICU for non-COVID patients/isolation ward without oxygen support/isolation ward with oxygen support/intubation kit/emergency and essential drugs/ambulance facility with ALS/separate functional OT for COVID emergencies/functional OT for non-COVID emergencies None
Health programs/schemes/projects ongoing	National health programs/Private/NGO schemes and projects (Government, NGO, and private)
Name of programs	Mobile Lab, Health Diary, Health Guide Health ID – Health Data Portal under the study None
Name of schemes	
Name of projects	
Does this facility have the necessary personal protective equipment for healthcare workers?	Yes, No
Whether the facility is willing to use/refer the following intervention under the ICMR Project:	
Location	
Image of the health facility (preferably front view)	

Appendix B. Laboratory Facilities

**Table 10:** Laboratory facilities

Question	Options/answers
Study site	Urban – Intervention (Kanhauli) Urban – Control (Balughat) Rural - Intervention (Mushahari) Rural - Control (Marwan) Health Facility Name
Laboratory name	
Laboratory type	
Laboratory address	
Contact number	
Name of the tests available	
Email	
Location	
Image	

Appendix C. Diagnostic Test Availability in the Laboratory Facilities

**Table 11:** Diagnostic test availability in the laboratory facilities CBC: complete blood count; KFT: kidney function test; LFT: liver function test; ESR: erythrocyte sedimentation rate; HIV: human immunodeficiency virus; HBsAg: hepatitis B surface antigen; HbA1C: glycosylated hemoglobin

Tests	Study Site				Total (N = 32)
	Marwan	Mushahari	Balughat	Kannauli	
CBC	3	4	12	9	28 (87.5%)
Blood glucose	3	4	12	9	28 (87.5%)
Lipid profile	3	3	12	9	27 (84.4%)
KFT	3	3	12	9	27 (84.4%)
LFT	3	3	12	9	27 (84.4%)
ESR	2	3	11	9	26 (81%)
Urine routine	2	3	11	8	25 (78%)
HIV	0	2	9	4	16 (50%)
HBsAg	0	2	8	4	14 (43.7%)
Widal	2	3	7	4	16 (50%)
Rapid malaria	2	3	8	4	17 (53%)
HbA1C	0	1	10	7	18 (56%)
Thyroid profile	0	1	9	5	15 (47%)
